# Transcriptome analysis reveals the link between lncRNA-mRNA co-expression network and tumor immune microenvironment and overall survival in head and neck squamous cell carcinoma

**DOI:** 10.1186/s12920-020-0707-0

**Published:** 2020-03-30

**Authors:** Zhaoming Zhong, Min Hong, Xiao Chen, Yan Xi, Yuanyuan Xu, Deyu Kong, Jun Deng, Yun Li, Rui Hu, Chuanzheng Sun, Jin Liang

**Affiliations:** 1 Department of Medical Oncology, First Affiliated Hospital of Kunming Medical University, Kunming Medical University, Kunming, China; 2 Department of Head and Neck Surgery Section II, Third Affiliated Hospital of Kunming Medical University, Kunming Medical University, Kunming, China; 3 Department of Oncology, First People’s Hospital of Kunming, Kunming, China

**Keywords:** HNSCC, lncRNA, Gene expression, Tumor microenvironment, Immune

## Abstract

**Background:**

As the sixth most common cancer worldwide, head and neck squamous cell carcinoma (HNSCC) develops visceral metastases during the advanced stage of the disease and exhibits a low five-year survival rate. The importance of tumor microenvironment (TME) in tumor initiation and metastasis is widely recognized. In addition, accumulating evidence indicates that long non-coding RNA (lncRNA) is involved in crosstalk between TME and tumor cells. However, the lncRNA-associated regulators modulating the HNSCC microenvironment and progression remain largely unknown.

**Methods:**

The publicly available transcriptome data and matched clinical HNSCC data were collected from The Cancer Genome Atlas (TCGA). Immune scores (ISs) and stromal scores (SSs) of HNSCC TME were calculated using ESTIMATE algorithm. Weighted gene co-expression network analysis (WGCNA) was conducted to determine the co-expressed lncRNAs and protein-coding mRNAs.

**Results:**

Results showed that the high IS HNSCC male patient subgroup exhibited improved survival. Additionally, we identified 169 lncRNAs and 825 protein-coding mRNAs that were differentially expressed in high IS HNSCC samples, with the up-regulated mRNAs displaying enrichment in immune-related biological processes. Notably, we identified a high co-expression lncRNA-mRNA module (i.e., purple module) that showed strong correlation with ISs. This module contained 79 lncRNAs and 442 mRNAs, including 26 lncRNAs and 215 mRNAs showing association between expression and male HNSCC survival. Consistently, 207 of the 215 mRNAs were up-regulated in high IS HNSCC group and were enriched in immune-related signaling pathways. Based on bioinformatics analyses and previous functional assays, certain lncRNAs (e.g., *AL365361.1* and *PCED1B-AS1*) in the purple module likely contributed to the modification of tumor immune microenvironment (TIME) in the high IS HNSCC patients, achieved by regulating transcription of abundant immune-related genes (e.g., *CCR7* and *TLR8*).

**Conclusions:**

In summary, we ascertained a HNSCC male patient subgroup that displayed high ISs and good survival probability. We identified hundreds of genes with specific expression patterns in this HNSCC subgroup as well as a highly co-expressed lncRNA-mRNA module with great potential for the modulation of TIME of HNSCC. Our study provides evidence of a link between the lncRNA-associated gene network, TIME, and HNSCC progression, and highlights potential therapeutic targets for this disease.

## Background

As one of the most common cancers worldwide, head and neck squamous cell carcinoma (HNSCC) currently accounts for approximately 600,000 cases each year [1, 2]. Most cases of HNSCC are comprised of malignancies arising in the oral cavity, oropharynx, hypopharynx, and larynx [1], with tobacco use and alcohol consumption reported to be important risk factors [3]. Despite advances in diagnosis and treatment strategies, the five-year survival rate of HNSCC patients remains below 50% [4], with locoregional invasion and metastasis identified as major causes of death [5]. However, the underlying molecular mechanisms of HNSCC pathogenesis remain poorly understood.

Communication between tumor cells and the surrounding microenvironment plays a crucial role in tumor growth, invasion, and metastasis [6, 7]. The tumor microenvironment (TME) mainly consists of host stromal cells, infiltrating immune cells, and extracellular matrix [8]. Accumulating evidence has identified various functions of these components in tumor development [7, 9, 10]. For instance, stromal fibroblasts are frequent components of tumor stroma and can produce growth factors (e.g., *TGF-1β*) that promote the initiation of cancers, including HNSCC [11–13]. Immune cells (e.g., absence of host CD8^+^ T cells) in the TME play important roles in modulating immune escape of tumor cells [9, 14]. As such, improving our understanding of HNSCC microenvironment dysregulation could provide novel opportunities for targeted disease therapy.

Currently, the close relationship between aberrant expression of non-coding RNAs (ncRNAs) and tumorigenesis is well established [15, 16]. Non-coding RNAs, as a class of RNAs without protein-coding function, are widely transcribed across the human genome [17]. Among them, long non-coding RNAs (lncRNAs), which exceed 200 nucleotides in length and do not encode proteins [18–20], participate in multiple biological processes, including cell proliferation and invasion [21, 22]. They are key regulators of cancer progression and modulate the transcription of cancer-related genes through chromatin modification and transcriptional processes [23–25]. To date, evidence has shown that some lncRNAs (e.g., *TSLNC8*, *MEG3*) function as tumor suppressors [26, 27], whereas others (e.g., *HOTAIR*, *PVT1*) serve as onco-lncRNAs [28, 29]. Recent reports also state that lncRNAs can function by mediating the TME [30, 31]; however, their potential regulatory roles in the TME and HNSCC progression remain unclear.

In the present study, we investigated the lncRNA-mediated networks that modulate the TME and are associated with survival in HNSCC patients. We downloaded and analyzed publicly available RNA-seq data from The Cancer Genome Atlas (TCGA) database, which included 500 HNSCC and 44 normal samples. We then assessed the stromal scores (SSs) and immune scores (ISs) of HNSCC patients using the ESTIMATE algorithm [32] and found that high ISs were associated with improved HNSCC outcome in male patients. In addition, we determined the differentially expressed genes (DEGs) between high and low IS HNSCC samples and identified a lncRNA-mRNA co-expression network with potential function in modulating the tumor immune microenvironment (TIME) and improving male HNSCC survival.

## Methods

### Data collection and preprocessing

The RNA-seq dataset (level 3), which consisted of 500 HNSCC and 44 normal tissue samples with clinical information, was obtained from the TCGA Data Portal (https://portal.gdc.cancer.gov/) using the GDC data transfer tool. A matrix of HTSeq read counts of genes was obtained. Among the 60,483 genes, we retained those with at least 10 reads in all samples (21,322 genes) for subsequent analyses. In addition, we also transformed the read counts of genes using the variance-stabilizing transformation (vst) algorithm from the ‘*vst*’ function in the DESeq2 package [33].

### Stromal and immune cell admixture analysis

The presence of stromal and immune cells in the TME of HNSCC samples was inferred using ESTIMATE (Estimation of Stromal and Immune cells in MAlignant Tumor tissues using Expression data) with gene expression signatures (vst-transformed gene read counts) of tumor samples [32].

### DEG analysis

The DEGs between groups were identified using the DESeq2 package based on the raw read counts of each gene [33], with a |log2FoldChange| threshold of > 1 and adjusted *P*-value threshold of < 0.05. During this process, the effect of gender was considered and corrected. The pheatmap package was used to draw the heatmap plot of DEGs.

### Functional and pathway enrichment analysis

Gene Ontology (GO) and Kyoto Encyclopedia of Genes and Genomes (KEGG) pathway enrichment analyses were performed using DAVID (v6.8) [34, 35]. A Benjamini-Hochberg (BH)-adjusted *P*-value cutoff of < 0.05 was used to determine the enriched biological processes and pathways. In addition, protein-protein interaction (PPI) analysis was performed using STRING (v11.0) with a minimum required interaction score of 0.4 (https://string-db.org/) [36].

### Survival analysis

Kaplan-Meier analysis was conducted to calculate the association between overall survival of HNSCC patients and various features (e.g., IS/SS, gene expression, and gender) using the R survival package. Samples were first separated into two subgroups according to the upper and lower quartile values of each feature. A log-rank test was used to calculate the *P*-value and the R survminer package was used to draw the survival curves. A *P*-value of < 0.05 was set as the threshold for statistical significance.

### lncRNA-mRNA co-expression analysis

We performed weighted gene co-expression network analysis (WGCNA) to determine the coding-non-coding gene interactions for the selected lncRNAs and mRNAs according to the protocols of the WGCNA R/Bioconductor package [37]. Automatic network construction with an optimal soft thresholding power of *β* = 8 was used. The ‘*exportNetworktoCytoscape’* function in the package with a threshold of 0.02 was used to generate the gene-gene interaction information. Cytoscape (v3.7.1; https://cytoscape.org/) was used to visualize the gene co-expression network.

## Results

### Clinical characteristics of HNSCC patients

We collected the RNA-seq profile data of 500 HNSCC and 44 normal samples with corresponding clinical information from the TCGA database (https://portal.gdc.cancer.gov/). The clinical characteristics of HNSCC patients are summarized in Additional file 1. The median age of patients was 61 years and the female to male ratio was ~ 1:2.8.

### Correlation between high ISs and improved survival in male HNSCC patients

**Fig. 1:**
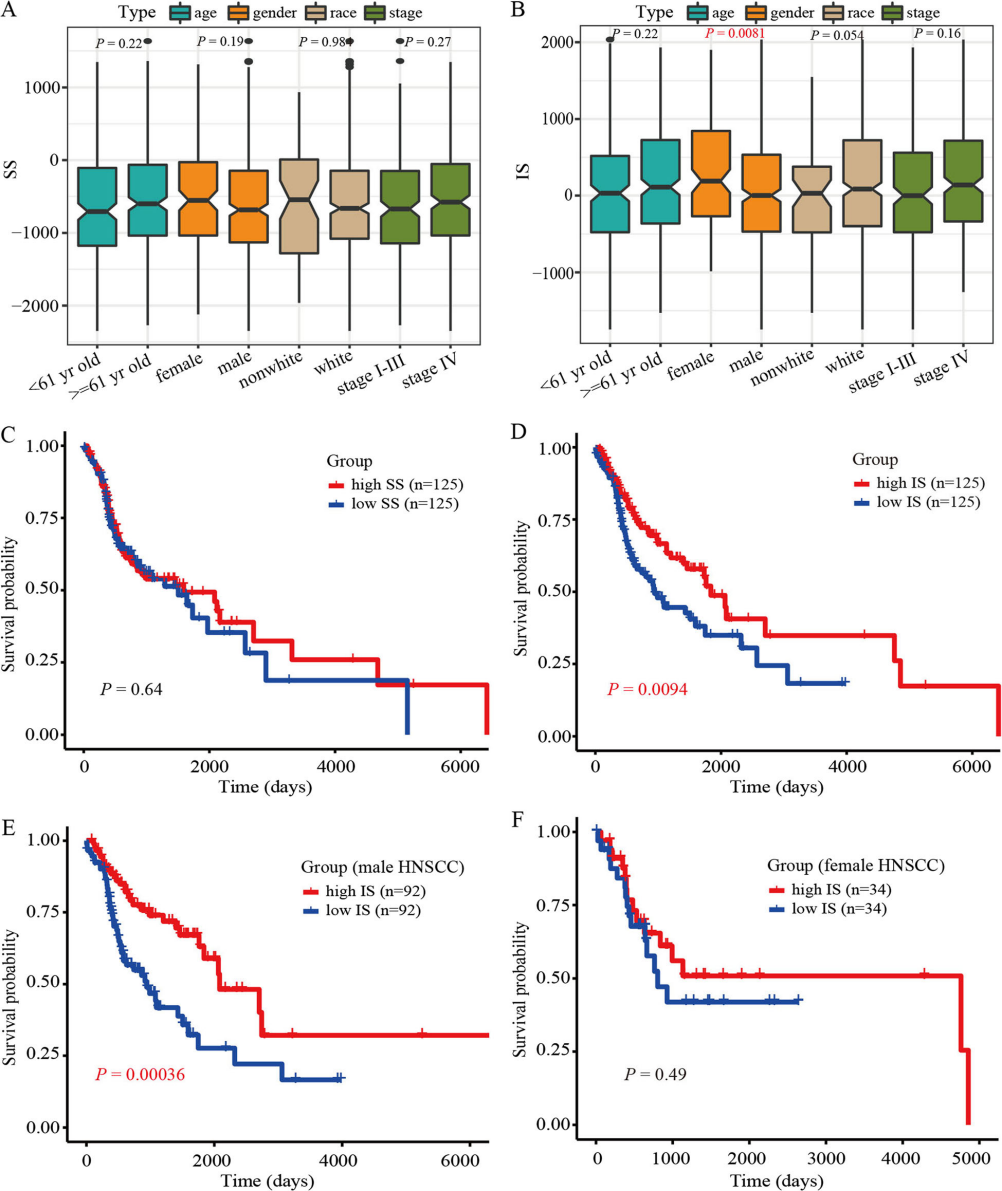
Immune scores (ISs) and stromal scores (SSs) associated with overall survival in head and neck squamous cell carcinoma (HNSCC) patients. **a** SSs in HNSCC with different clinical features. **b** ISs in HNSCC with different clinical features. **c** Kaplan-Meier survival curves displayed no association between SSs and HNSCC survival. **d** Kaplan-Meier survival curves revealed a significant correlation between ISs and HNSCC survival. High IS HNSCC samples (red color) showed good survival probability. **e**-**f** Kaplan-Meier survival analyses showed significant association between ISs and HNSCC survival in male (but not female) patients

We inferred the infiltration of non-tumor cells in the TME of HNSCC based on gene expression data using ESTIMATE [32]. For the HNSCC patients, the SSs ranged from − 2349.5 to 1635.3 and ISs ranged from − 1745.8 to 2034.8 (Additional file 2). Furthermore, the HNSCC samples had higher SSs and ISs than that of the normal samples (*P* < 0.05; Additional file 2). The ISs and SSs showed no significant differences between patients based on age, race, or stage (*P* > 0.05), although ISs (but not SSs) showed significant differences between female and male HNSCC patients (*P* < 0.05, Fig. 1a and b).

To test whether the SSs and/or ISs were significantly associated with survival in HNSCC subjects, we performed Kaplan-Meier survival analyses and found a significant association between ISs and HNSCC survival, with the high IS HNSCC samples exhibiting good survival probability (*P* = 0.0094, Fig. 1c and d). As the ISs of HNSCC patients were gender-biased and high scores were markedly associated with improved survival in male (but not female) HNSCC patients (*P* = 0.00036, Fig. 1e and f). In addition, we also conducted three random resampling of 133 male HNSCC patients (i.e., equal to the number of female samples) for survival analysis and obtained stable results (Additional file 3). Therefore, we focused on the association between these scores and survival in male HNSCC patients.

### DEGs between HNSCC male patient subgroups with high or low ISs

**Fig. 2:**
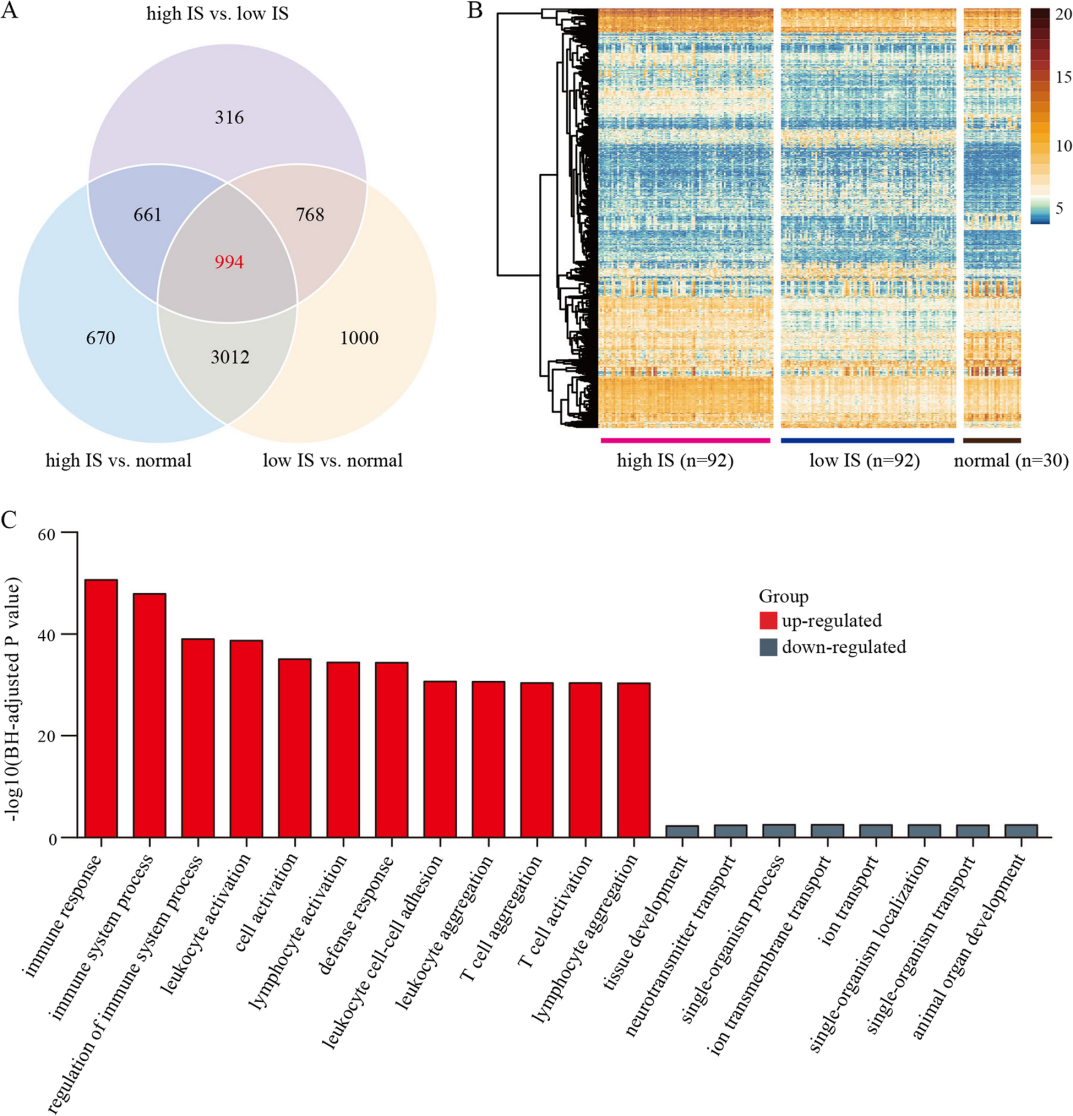
Differentially expressed genes (DEGs) between high and low HNSCC subgroups in male patients. **a** Venn plot of DEGs. Intersection of datasets contained TIME-associated genes. **b** Heatmap plot displayed expression differences in DEGs between high and low IS HNSCC patients and normal samples. Transformed read counts of genes using the variance-stabilizing transformation (vst) algorithm were used to represent gene expression in each sample. **c** Based on GO analysis, up-regulated protein-coding genes in high IS HNSCC subgroup were significantly enriched in immune-related biological processes. Y-axis represents negative logarithm to base 10 of the *P*-value

We first identified a total of 2739 DEGs between the high and low IS HNSCC subgroups, consisting of 184 samples (Table 1). In addition, 994 of these DEGs also displayed differences in the high IS HNSCC samples compared with the low IS HNSCC and normal samples (Fig. 2a and Additional file 4), as visualized in the heatmap plot (Fig. 2b). We defined these 994 DEGs as TIME-related genes, which included 169 lncRNAs and 825 protein-coding mRNAs. Among them, 48 and 121 lncRNAs and 579 and 246 protein-coding mRNAs were up-regulated and down-regulated, respectively, in the high IS HNSCC samples.

**Table 1:** Clinical information on high and low IS HNSCC samples

Clinical variable	High IS HNSCC	Low IS HNSCC
Number	92	92
Age (years)	61.5 ± 10.8	58.8 ± 11.0
Gender	male	male
IS range	537.8–2034.8	−1745.3–-473.5
Clinical stage		
Stage I-III	34	41
Stage IV	56	46
Unknown	2	5
Race		
White	84	72
Other	8	20
Vital status		
Alive	62	43
Dead	30	39

To further investigate the functions of TIME-related genes, we performed functional analysis of the protein-coding mRNAs. In line with the above findings, GO analysis showed that the up-regulated genes in the high IS HNSCC subgroup were significantly enriched in immune-related biological processes (e.g., immune response, regulation of immune system process, leukocyte activation, T cell aggregation, and T cell activation), whereas the down-regulated genes were enriched in processes involved in ion transportation (e.g., cation transport and ion transmembrane transport) (Fig. 2c), suggesting an important role of up-regulated genes in the modulation of HNSCC TIME.

### Co-expressed lncRNA-mRNA network associated with ISs in male HNSCC samples

**Fig. 3:**
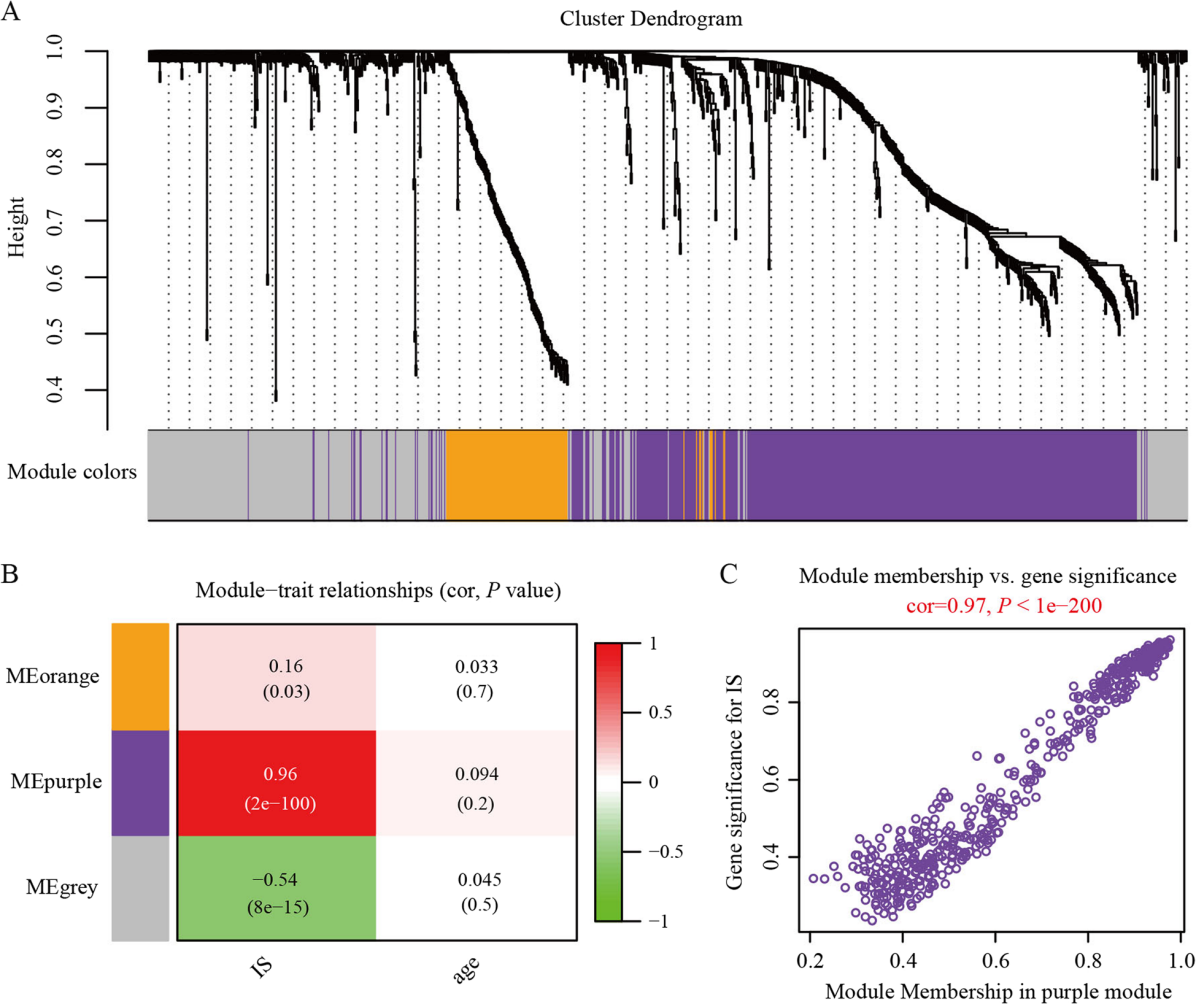
Two lncRNA-mRNA co-expression modules in male HNSCC patients. **a** Cluster dendrogram of connected genes produced two lncRNA-mRNA co-expression modules (i.e., purple and orange). Genes not assigned into one of the two modules are labeled in gray. **b** Heatmap of each cell containing *P-*value and correlation coefficient for each module and trait of interest (rows correspond to module; columns correspond to HNSCC traits of interest). **c** Scatterplot of module membership and gene significance showing strong correlation between purple module and ISs

Considering the potential transcription regulatory functions of lncRNAs and mRNAs [38, 39], we constructed and analyzed the coding-non-coding gene co-expression network of the 994 TIME-associated genes using WGCNA [37]. We identified two co-expressed gene modules, which are labeled with different colors (i.e., purple and orange) in Fig. 3a. Results showed that the purple module contained 521 genes and orange module contained 127 genes.

We then calculated the correlation coefficients between modules and traits of interest, with the purple module exhibiting strong correlation with ISs (correlation coefficient = 0.96, Fig. 3b). In addition, the correlated gene significance and module membership were plotted for the purple module, again demonstrating that genes in this module were significantly associated with ISs (Fig. 3c). Taken together, these findings indicate that genes in the purple module had marked effects on the dynamic ISs of HNSCC patients, especially those of males.

### TIME-associated genes in purple module associated with male HNSCC survival

**Fig. 4:**
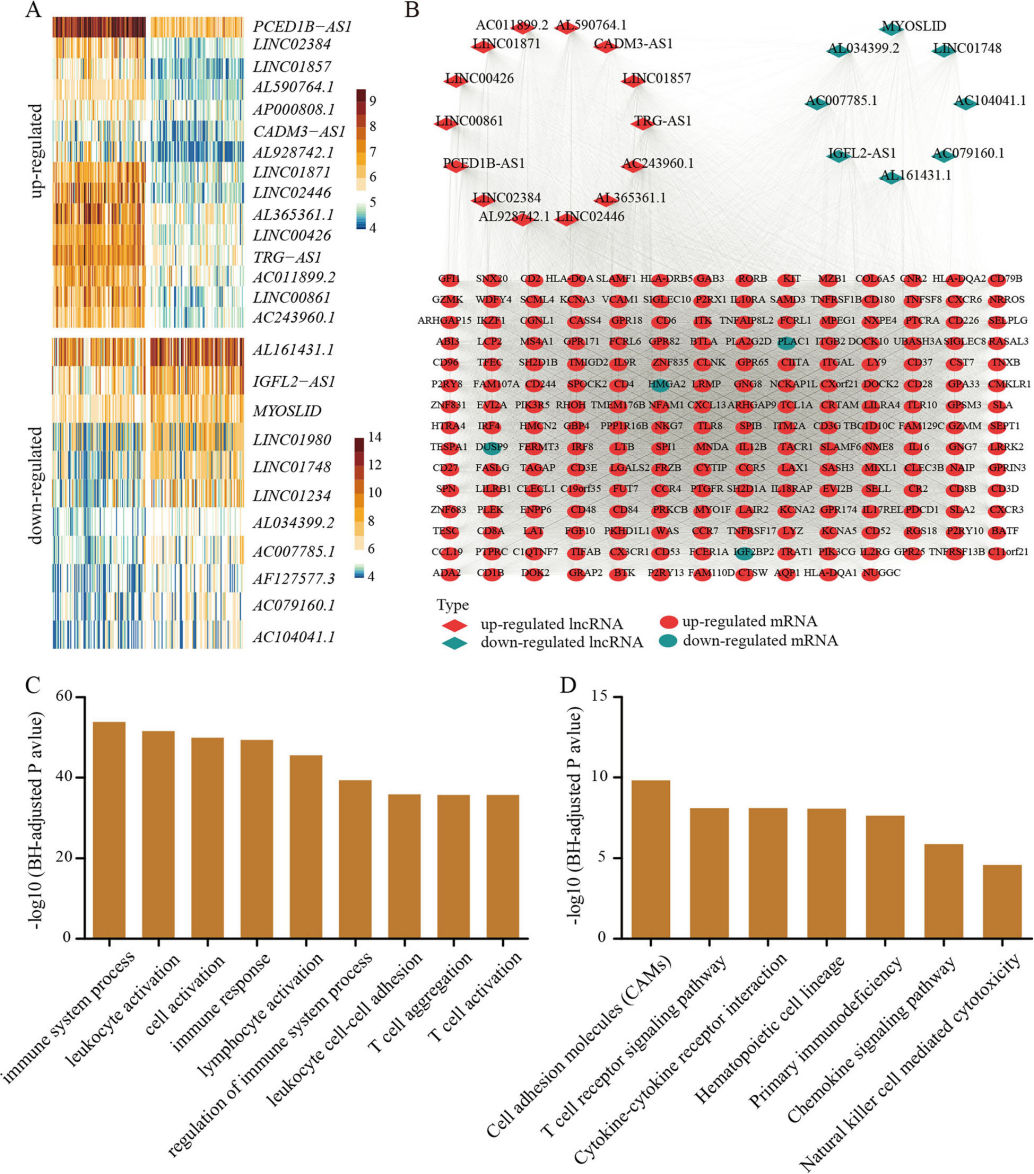
TIME-related lncRNA-mRNA co-expression network associated with overall survival in male HNSCC patients. **a** Heatmap plot for 26 lncRNAs in purple module associated with male HNSCC survival. **b** Networks of lncRNAs and mRNAs in purple module. Only gene pairs associated with male HNSCC survival are shown in network plot. Genes were connected by an edge if correlation between their expression was significant. **c** GO analysis showed that up-regulated mRNAs associated with male HNSCC survival in purple module were enriched in immune-related biological processes. **d** KEGG analysis revealed that up-regulated mRNAs associated with male HNSCC survival in purple module were overrepresented in multiple immune-related pathways

We subsequently focused on the purple module as it displayed the strongest correlation with ISs (Fig. 3b and c). This module consisted of 79 lncRNAs and 442 mRNAs. To investigate the effects of TIME-associated lncRNA/mRNA expression on survival of male HNSCC patients, we separated the samples into two subgroups according to the upper and lower quartiles of expression levels of each TIME-associated lncRNA/mRNA and performed survival analysis. In total, we identified 26 lncRNAs and 215 mRNAs with significant associations between expression and male HNSCC survival (*P* < 0.05), including 15 and 11 up- and down-regulated lncRNAs and 207 and 8 up- and down-regulated protein-coding mRNAs, respectively, in the high IS HNSCC samples (Fig. 4a and Additional file 5). For the purple module, those genes displaying co-expression patterns and associated with male HNSCC survival are shown in Fig. 4b (Additional file 6).

Furthermore, based on GO analysis, the 207 up-regulated TIME-related mRNAs in the purple module again showed greatest enrichment in immune-related biological processes, such as immune response, leukocyte activation, and T cell activation (Fig. 4c). Consistently, KEGG pathway analysis revealed that these genes were enriched in the T cell receptor signaling pathway, cytokine-cytokine receptor interaction, and chemokine signaling pathway (Fig. 4d). We also conducted PPI analysis and revealed a high degree of interaction among the up-regulated protein-coding mRNAs in the high IS HNSCC samples (*P* < 1.0e-16), with significant enrichment in immune-related biological processes (Additional file 7). Thus, the highly co-expressed lncRNAs-mRNA networks identified in this study likely play important roles in regulating the TIME and in modulating male HNSCC survival.

## Discussion

The TME participates in cancer progression, including that of HNSCC [7, 40, 41]. Understanding the molecular mechanisms involved in tumor-induced immune suppression and developing effective strategies to reverse the suppressive TME should facilitate improvement in the survival of HNSCC patients. To date, numerous studies have revealed the close relationship between gene transcription and the TME [42, 43]; however, TME-associated gene expression characteristics and underlying regulatory mechanisms of HNSCC remain to be investigated.

In this study, we calculated the SSs and ISs of HNSCC samples using ESTIMATE [32] based on the transcriptome data of 500 HNSCC and 44 normal samples from the TCGA. Interestingly, we found that ISs, as an index of the TIME, were positively associated with overall survival probability in male HNSCC patients. In line with this, based on the DEGs (i.e., 169 lncRNAs and 825 mRNAs) between the HNSCC male subgroups, the up-regulated protein-coding genes in the high IS HNSCC subgroup showed significant enrichment in immune-related biological processes, such as lymphocyte and T cell activation. These findings indicate that the specific transcription profiles in the high IS HNSCC subgroup, at least in male patients, likely contribute to their improved survival probability via modulation of the TIME.

**Fig. 5:**
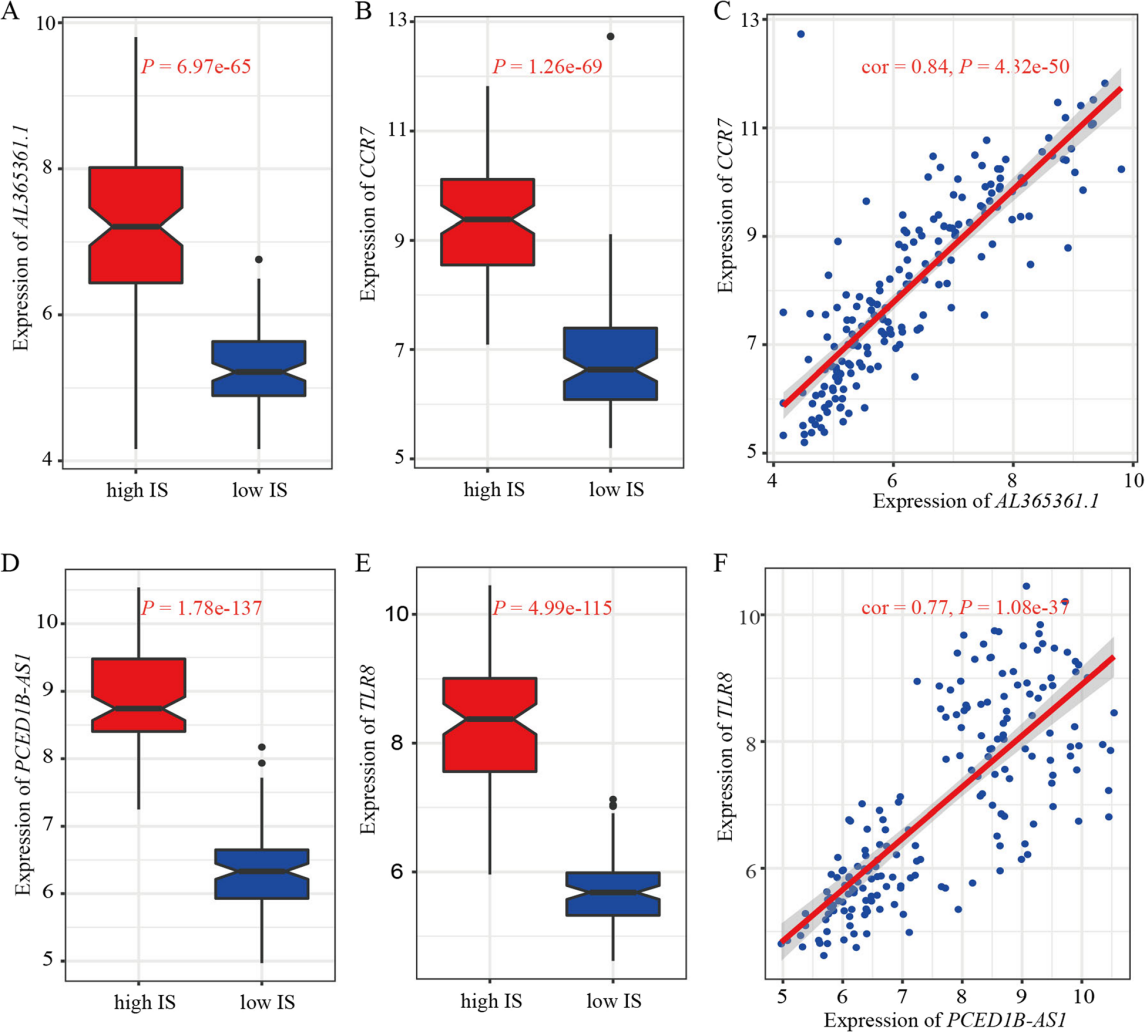
Roles of TIME-lncRNAs in improving outcome in male HNSCC patients. **a** Up-regulation of *AL365261.1* in the high IS HNSCC samples. **b** Up-regulation of *CCR7* in the high IS HNSCC samples. **c** A positive correlation found in the expression levels between *AL365261.1* and *CCR7*. **d** Up-regulation of *PCED1B-AS1* in the high IS HNSCC samples. **e** Up-regulation of *TLR8* in the high IS HNSCC samples. **f** A positive correlation found in the expression levels between *PCED1B-AS1* and *TLR8*

Accumulating evidence suggests that lncRNAs play important roles in the regulation of immune-related processes [44, 45]. In addition, it is widely recognized that lncRNAs are important regulatory factors in modulating the TIME and tumor progression [30, 46]. Therefore, we constructed a functional lncRNA-mRNA co-expressed network using genes exhibiting expression differences between the high and low IS HNSCC male subgroups. Of note, we identified a lncRNA-mRNA co-expression module (i.e., purple module) that showed significant association with ISs. Indeed, hundreds of lncRNAs/mRNAs in this module demonstrated associations with male HNSCC survival, with the up-regulated protein-coding mRNAs enriched in multiple immune processes, implying that genes in this module possibly control HNSCC TIME and survival. In addition, we examined several detailed cases to further confirm the roles of the lncRNA-mRNA regulatory connections in the suppression of HNSCC development. For instance, *AL365361.1* was upregulated in the high IS HNSCC samples and positively associated with *CCR7* expression (Fig. 5a-c). Earlier studies have implicated *AL365361.1* in the prediction of early hepatocellular carcinoma recurrence and in the modulation of malignant progression in ovarian cancer [47, 48]. In addition, previous research has revealed that tumor infiltration by T lymphocytes with increased *CCR7* expression is associated with favorable outcome in cancer patients [49]. We also found that *PCED1B-AS1* was up-regulated in the high IS HNSCC samples and positively associated with *TLR8*, a gene-encoding Toll-like receptor (Fig. 5d-f). Prior study has revealed that *TLR8* can prevent T-cell senescence and further enhance tumor immunosuppressive function [50]. From the corresponding protein-coding genes, we found these two lncRNAs to be located in different chromosomes, indicating possible transregulatory mechanisms for the lncRNA-mRNA pairs. Overall, these findings suggest that the identified lncRNA-mRNA co-expression module likely plays an important role in modulating the TIME and improving overall survival in male HNSCC patients.

## Conclusions

In summary, our study suggests that the dynamic expression of genes related to immune biological processes has a marked influence on the TIME and HNSCC outcome, at least in male patients. Importantly, we identified a highly co-expressed lncRNA-mRNA network that likely plays an important role in improving overall HNSCC survival in male patients via alteration of the TIME. Nevertheless, further experimental and clinical studies are required to shed additional light on the function of the genes in this lncRNA-mRNA co-expression network on HNSCC progression.

## Supplementary information

**Additional file 1.** Summary information on HNSCC samples.

**Additional file 2.** Immune scores (ISs) and stromal scores (SSs) of head and neck squamous cell carcinoma (HNSCC) samples. (A) Ranges of ISs and SSs in HNSCC patients. (B) Differences in ISs and SSs between HNSCC patients and normal samples.

**Additional file 3.** Association between ISs and male HNSCC survival with three random resampling of 133 male patients.

**Additional file 4.** List of the DEGs in high IS HNSCC subgroups compared to low IS HNSCC and normal samples.

**Additional file 5.** Information of the genes in purple module that associated with male HNSCC survival.

**Additional file 6.** Information of the gene-gene interactions in purple module.

**Additional file 7.** Protein-protein interaction (PPI) network of up-regulated protein-coding mRNAs in high IS HNSCC subgroup.

## Data Availability

The HNSCC RNA-seq data were downloaded from the TCGA (https://portal.gdc.cancer.gov/projects/TCGA-HNSC).

## Abbreviations

HNSCC: head and neck squamous cell carcinoma
TCGA: The Cancer Genome Atlas
TME: Tumor microenvironment
TIME: Tumor immune microenvironment
ESTIMATE: Estimation of Stromal and Immune cells in MAlignant Tumor tissues using Expression data
SS: Stromal score
IS: Immune score
GO: Gene Ontology
KEGG: Kyoto Encyclopedia of Genes and Genomes
PPI: Protein-protein interaction
WGCNA: Weighted gene co-expression network analysis
