# Supplementary material for: Transcriptome analysis reveals the link between lncRNA-mRNA co-expression network and tumor immune microenvironment and overall survival in head and neck squamous cell carcinoma
Source: BMC Med Genomics. 2020 Mar 30;13:57. doi: 10.1186/s12920-020-0707-0 (PMC7104528; doi:10.1186/s12920-020-0707-0)
Supplement: Supplementary file 1 — Additional file 1. Summary information on HNSCC samples. [file 12920_2020_707_MOESM1_ESM.doc]

Additional file 1. Summary information on HNSCC samples.

| Parameter | Subtype | Number of patients (%) |
| --- | --- | --- |
| Age (years) | >=61 | 255 (51%) |
|  | <61 | 244 (48.8%) |
| Gender | female  male | 133 (26.6%)  367 (73.4) |
| Vital status | alive  dead | 282 (56.4%)  218 (43.6%) |
| Race | white  nonwhite | 426 (85.2%)  74 (14.8%) |
| Tumor stage | stage I-III  stage IV | 173 (34.6%)  259 (51.8%) |
