# Supplementary material for: Transcriptome analysis reveals the link between lncRNA-mRNA co-expression network and tumor immune microenvironment and overall survival in head and neck squamous cell carcinoma
Source: BMC Med Genomics. 2020 Mar 30;13:57. doi: 10.1186/s12920-020-0707-0 (PMC7104528; doi:10.1186/s12920-020-0707-0)

Additional file 2. Immune scores (ISs) and stromal scores (SSs) of head and neck squamous cell carcinoma (HNSCC) samples. (A) Ranges of ISs and SSs in HNSCC patients. (B) Differences in ISs and SSs between HNSCC patients and normal samples.


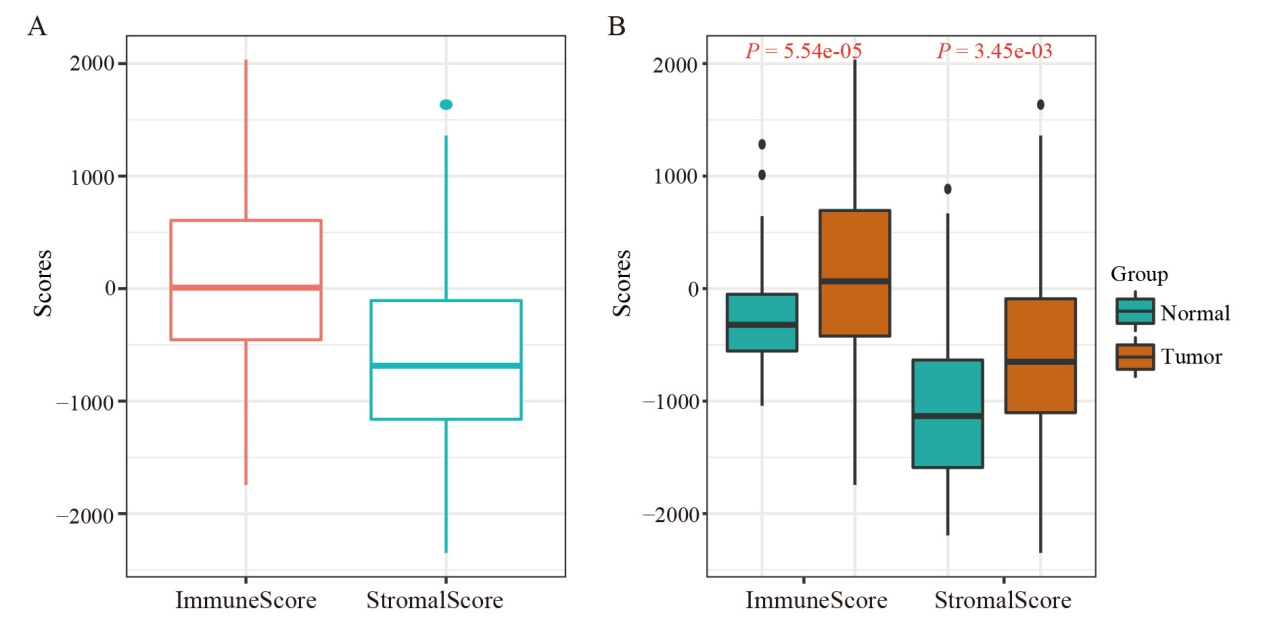

Supplement: Supplementary file 2 — Additional file 2. Immune scores (ISs) and stromal scores (SSs) of head and neck squamous cell carcinoma (HNSCC) samples. (A) Ranges of ISs and SSs in HNSCC patients. (B) Differences in ISs and SSs between HNSCC patients and normal samples. [file 12920_2020_707_MOESM2_ESM.docx]
