# Supplementary material for: Transcriptome analysis reveals the link between lncRNA-mRNA co-expression network and tumor immune microenvironment and overall survival in head and neck squamous cell carcinoma
Source: BMC Med Genomics. 2020 Mar 30;13:57. doi: 10.1186/s12920-020-0707-0 (PMC7104528; doi:10.1186/s12920-020-0707-0)

Additional file 3. Association between ISs and male HNSCC survival with three random resampling of 133 male patients.


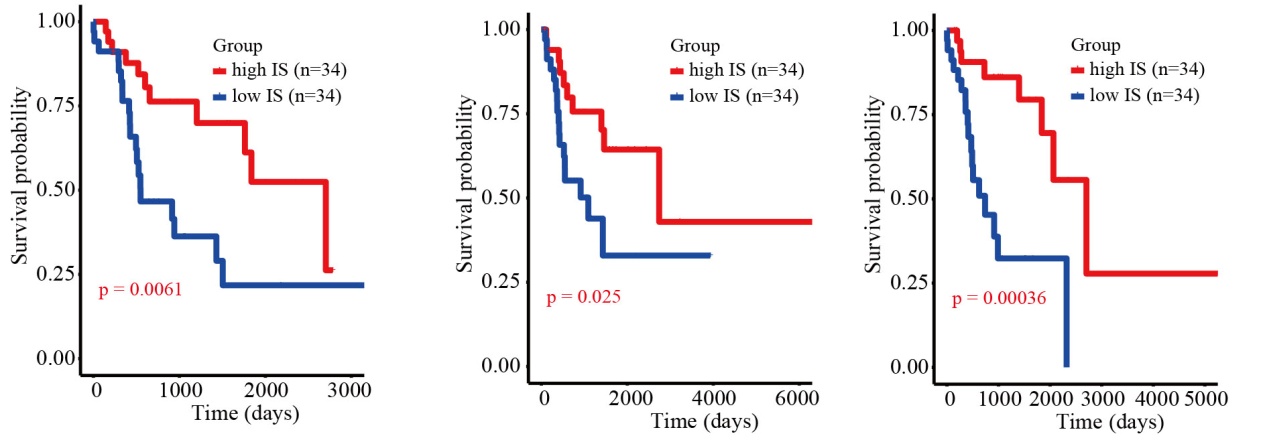

Supplement: Supplementary file 3 — Additional file 3. Association between ISs and male HNSCC survival with three random resampling of 133 male patients. [file 12920_2020_707_MOESM3_ESM.docx]
