# Supplementary material for: Transcriptome analysis reveals the link between lncRNA-mRNA co-expression network and tumor immune microenvironment and overall survival in head and neck squamous cell carcinoma
Source: BMC Med Genomics. 2020 Mar 30;13:57. doi: 10.1186/s12920-020-0707-0 (PMC7104528; doi:10.1186/s12920-020-0707-0)

Additional file 7. Protein-protein interaction (PPI) network of up-regulated protein-coding mRNAs in high IS HNSCC subgroup. Bioinformatics analysis revealed that these genes were significantly overrepresented in immune-related biological processes.


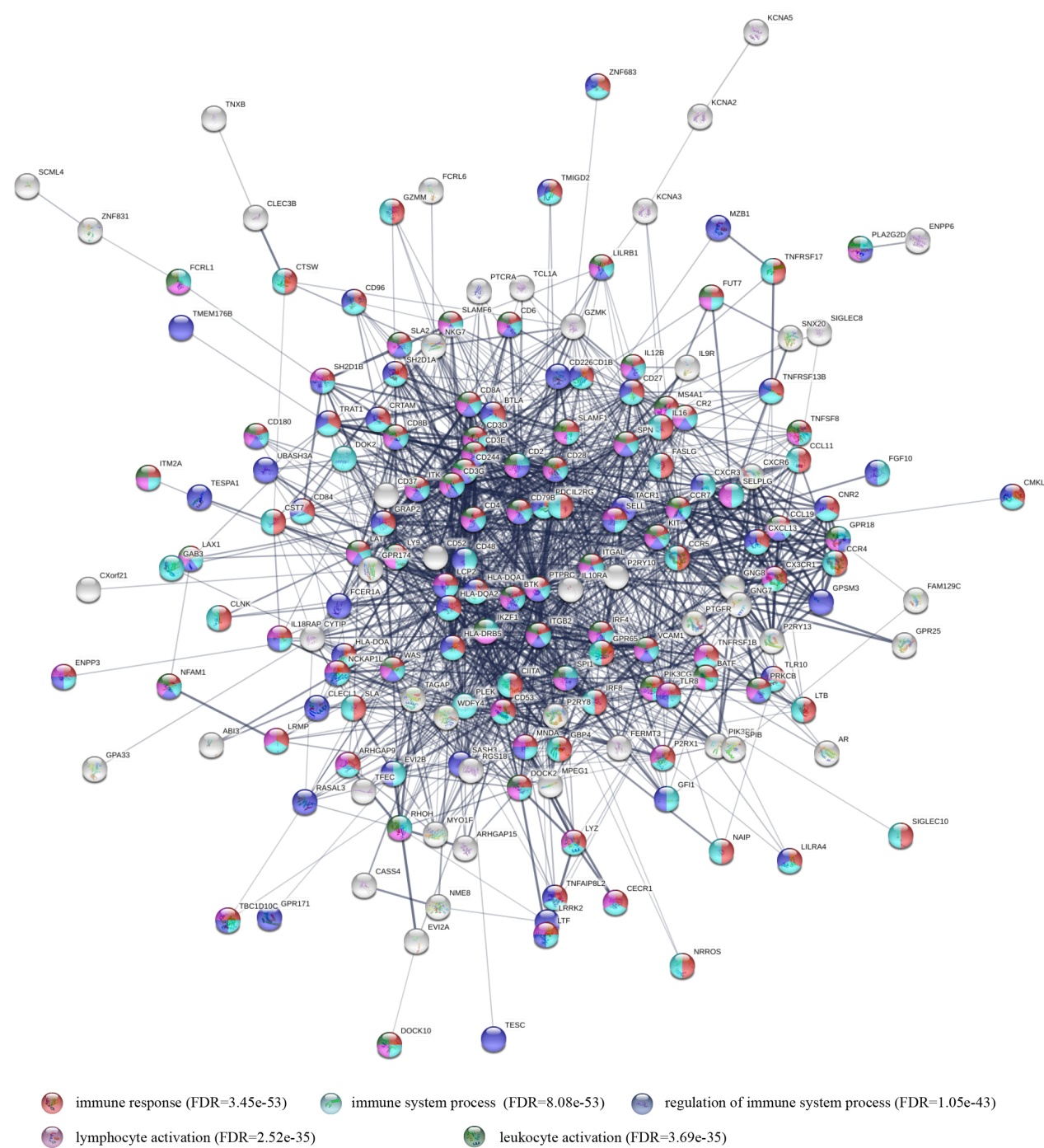

Supplement: Supplementary file 7 — Additional file 7. Protein-protein interaction (PPI) network of up-regulated protein-coding mRNAs in high IS HNSCC subgroup. [file 12920_2020_707_MOESM7_ESM.docx]
